# P-70. A Comparison of Clinical and Safety Outcomes for Patients with Vancomycin Resistant Enterococcus Faecium Bacteremia Treated with High Dose Daptomycin Based on Isolate MIC

**DOI:** 10.1093/ofid/ofaf695.299

**Published:** 2026-01-11

**Authors:** Taylor Wisdom, Marco R Scipione

**Affiliations:** Detroit Medical Center, Detroit, Michigan; Detroit Receiving Hospital, Detroit, MI

## Abstract

**Background:**

Daptomycin (DAP) is a frequently utilized treatment option for patients with vancomycin-resistant Enterococci (VRE) bacteremia. Prior study data has shown poorer clinical outcomes for patients with VRE bacteremia and isolate MICs of >1µg/mL who were treated with standard dosing DAP (6mg/kg/day). The purpose of this project is to compare clinical and safety outcomes among patients with VRE faecium bacteremia and an isolate MIC of 2-4 µg/mL based on daptomycin dose.

**Methods:**

This is a retrospective chart review assessing outcomes for patients ≥18yo treated with DAP for VRE bacteremia. Patients were excluded if they had concurrent treatment with a VRE active agent, were pregnant, had a primary infectious focus of pneumonia. Primary outcome is 14-day mortality based on DAP dose used (< 10mg/kg vs ≥10mg/kg) for isolates with an MIC of 2 µg/mL or 4µg/mL. Secondary outcomes include analysis of 14-day mortality based on both DAP dose used and isolate MIC, hospital mortality, time to microbiological clearance, microbiological failure, and clinical failure.

**Results:**

95 pts with VRE bacteremia were included. There were 53 pts in the high DAP group (< 10mg/kg) and 42 pts in the low DAP dose group (≥10mg/kg). Baseline and treatment characteristics were similar between groups. 14-day mortality was low overall and not significantly different between groups (7% vs. 6%, p=0.72). There were also no differences between in-hospital mortality, microbiological failure, and clinical failure. There were no differences in adverse events. Median time to microbiological clearance was faster in the high-dose DAP group (2 [2-4] vs. 3 [2.5-5] days, p=0.02). There were no differences in outcomes between groups depending on MIC; however time to microbiological clearance was significantly lower in the high-dose DAP group when MIC was 4 µg/mL (2 [2-5] v. 3 [2-6], p=0.04)

**Conclusion:**

Low-dose compared to high-dose DAP was not associated with 14-day mortality. Low-dose DAP was not significantly associated with overall hospital mortality, microbiological failure, or clinical failure. High-dose DAP was associated with decreased time to microbiological clearance. High-dose DAP dosing does not appear to be associated with an increased risk of adverse drug events compared to low-dose.

**Disclosures:**

All Authors: No reported disclosures

